# Colonization of Endophytic *Bacillus velezensis* BHZ-29 in Cotton and Its Induction of Resistance to Cotton Verticillium Wilt

**DOI:** 10.3390/microorganisms14071600

**Published:** 2026-07-22

**Authors:** Yingwu Shi, Xinxiang Niu, Ablimit Nuraliya, Yue Sheng, Hongmei Yang, Min Chu, Ning Wang, Huifang Bao, Kai Lou

**Affiliations:** 1 Institute of Microbiology, Xinjiang Academy of Agricultural Sciences, Urumqi 830091, China; 2 Xinjiang Laboratory of Special Environmental Microbiology, Urumqi 830091, China; 3 Key Laboratory of Agricultural Environment in Northwest Oasis of Ministry of Agriculture and Countryside, Urumqi 830091, China; 4 Institute of Agricultural Resources and Environment, Xinjiang Academy of Agricultural Sciences, Urumqi 830091, China

**Keywords:** biological control, endophytic bacteria, induced systemic resistance, plant–pathogen interaction, sustainable agriculture

## Abstract

Cotton Verticillium wilt is a devastating fungal disease caused by *Verticillium dahliae*, and biological control has become a safe and efficient strategy for its green prevention and control. In this study, the endophytic strain *Bacillus velezensis* BHZ-29 was used as the biocontrol material, and rifampicin labeling and greenhouse pot assays were performed to clarify its colonization characteristics and induced disease resistance mechanism in cotton. The results showed that the rifampicin-resistant mutant strain maintained consistent morphological traits, antagonistic activity and biocontrol performance with the wild-type strain, ensuring the reliability of colonization tracing. Strain BHZ-29 could stably colonize the roots, stems and leaves of different cotton varieties, with roots serving as the dominant colonization tissue. Physiological analysis indicated that BHZ-29 inoculation significantly activated the antioxidant and defense enzyme system of cotton, including POD, CAT, SOD, PPO and PAL. The defense enzyme activities of cotton leaves showed a trend of first increasing and then decreasing, and the combined inoculation of BHZ-29 and *V. dahliae* exhibited the highest enzyme activity. Meanwhile, BHZ-29 treatment significantly increased vitamin C content and reduced malondialdehyde accumulation in cotton, alleviating pathogen-induced oxidative damage. Field pot verification confirmed that BHZ-29 possessed excellent and broad-spectrum biocontrol effects on cotton Verticillium wilt with stable control efficacy across different cotton varieties. This study clarifies the colonization and induced resistance mechanism of strain BHZ-29 against Verticillium wilt, providing a promising microbial resource and theoretical basis for the green biological control of cotton soil-borne diseases.

## 1. Introduction

Cotton is one of the most important economic crops in China, playing a crucial role in the agricultural sector. However, its production is severely threatened by Verticillium wilt, a devastating disease caused by the pathogen Verticillium dahliae [1]. This disease has significantly hindered the growth and development of the cotton industry. Verticillium dahliae Kleb. is notorious for its wide host range and diverse modes of transmission, complicating control efforts [2]. The pathogen’s persistence is further exacerbated by microsclerotia, which can remain viable in the soil for extended periods, perpetuating the infection cycle [3,4].

In recent years, the principles of sustainable development and green agriculture have gained widespread acceptance. Consequently, biological control has emerged as a promising strategy for managing crop diseases, including Verticillium wilt. This approach focuses on using efficient antagonistic bacteria to suppress pathogens [4,5,6]. Traditionally, the selection of biocontrol agents has relied on the plate confrontation method. However, the effectiveness of strains identified through this method may vary in real-world applications due to multiple factors. The success of biocontrol largely depends on the interactions among host plant characteristics, pathogens, environmental conditions, and the properties of the biocontrol agents themselves [7].

A critical factor for effective biocontrol is the ability of antagonistic bacteria to colonize the plant and adapt to adverse environmental conditions. Stable colonization is essential for the bacteria to exert antagonistic effects and enhance overall biocontrol efficacy. Malondialdehyde (MDA) can crosslink and polymerize with membrane proteins and enzymes, thereby disrupting the structure and function of cell membranes, which results in increased membrane permeability. The antioxidant system of cotton, including vitamin C, is influenced by biocontrol bacteria, effectively regulating the levels of reactive oxygen species. Consequently, the degree of membrane lipid peroxidation is reduced, and MDA accumulation is minimized. This helps maintain the integrity of the cell membrane, enabling better resistance against bacterial invasion and toxin damage. Furthermore, plant disease resistance is often associated with the activity of various defense-related enzymes, such as polyphenol oxidase (PPO), phenylalanine ammonia-lyase (PAL), superoxide dismutase (SOD), peroxidase (POD), and catalase (CAT) [8,9,10]. Numerous studies have demonstrated that biocontrol bacteria can induce changes in these enzymes, thereby enhancing plant disease resistance [11].

For instance, root irrigation with Bacillus subtilis TR21 fermentation broth increased the activities of POD, PPO, and PAL in banana roots compared to the control [12]. Similarly, Chen et al. [13] reported that the fermentation broth and supernatant of Bacillus subtilis CS16 could induce changes in the activities of SOD, PPO, PAL, and POD in banana leaves.

Despite these advances, two critical gaps remain largely unaddressed. First, most current studies focus on the in vitro antagonistic activity or the final disease reduction effect, while systematic investigations into the in vivo colonization dynamics of biocontrol strains—and their direct correlation with induced systemic resistance—are still scarce. Second, although various biocontrol bacteria have been shown to modulate defense enzyme activities, the specific mechanisms by which a newly isolated strain influences both membrane lipid peroxidation (measured via MDA and Vc) and a comprehensive panel of defense enzymes (POD, PPO, PAL, CAT) in cotton against *V. dahliae* have not been concurrently explored. Addressing these gaps is essential for the rational design and application of biocontrol agents under field conditions.

Our laboratory previously isolated the BHZ-29 strain from cotton plants in Bole, which demonstrated strong antagonistic effects against *V. dahliae* in vitro [14]. However, whether BHZ-29 can successfully colonize cotton tissues in vivo and how its colonization affects the cotton defense enzyme system and oxidative stress responses have not yet been reported. Therefore, the novelty of this work lies in its integrated approach: we simultaneously evaluated the colonization ability of BHZ-29 in cotton roots, stems, and leaves and systematically measured the temporal changes in POD, PPO, PAL, and CAT activities, as well as MDA and vitamin C contents, under *V. dahliae* challenge. This integrated investigation not only extends the current understanding of BHZ-29 beyond its preliminary antagonistic activity but also provides, for the first time, mechanistic insights into its role in inducing systemic resistance. The findings are expected to provide a theoretical basis for developing and applying this biocontrol bacterium in sustainable cotton production.

## 2. Materials and Methods

### 2.1. Test Strains and Cotton Varieties

*Bacillus velezensis* BHZ-29 [14], effective against cotton Verticillium wilt, was isolated from cotton root tissue by our laboratory in Bole City, Xinjiang. *V. dahliae* was provided by Liu Haiyang from the Institute of Plant Protection, Xinjiang Academy of Agricultural Sciences. The cotton cultivars used in this study—Xinluzao 36S (susceptible), Xinluzao 61T, and 9T (disease-resistant)—were obtained from the Shihezi Academy of Agricultural Sciences.

### 2.2. Screening of Antibiotic-Resistant Mutant Strains

A rifampicin stock solution was prepared by accurately weighing 2.5 g of rifampicin, dissolving it in dimethyl sulfoxide (DMSO), and then adding sterile water to a final volume of 50 mL after complete dissolution. The solution was filter-sterilized through a 0.22 μm membrane to obtain a 50 mg/mL stock solution, which was stored at −20 °C. Antagonistic bacteria were transferred to nutrient agar (NA) plates containing 10 μg/mL rifampicin and incubated at 34 °C for 24 h. Mutant strains exhibiting robust growth were selected and subcultured on NA plates with the same rifampicin concentration. After two passages, they were transferred to NA plates containing 20 μg/mL rifampicin. This process was repeated, gradually increasing the rifampicin concentration, until mutant strains capable of normal growth on NA plates with 300 μg/mL rifampicin were obtained. These strains displayed colony morphology identical to the original antagonistic bacteria and maintained unchanged antagonistic effects against the pathogen [15,16].

### 2.3. Genetic Stability and Resistance Activity Detection of RIF-Resistant B. velezensis Strains

A single colony of the labeled strain was inoculated into 50 mL of nutrient broth (NB) medium in a 250 mL flask and shaken at 32 °C and 180 rpm for 6 h, representing the first generation of rifampicin (RIF)-resistant strains. The bacterial suspension was then transferred at a 1% inoculum (0.5 mL, 4.52 × 10^8^ CFU/mL) to fresh NB medium and cultured under the same conditions to obtain subsequent generations. This subculturing process was repeated for 30 generations. Bacterial suspensions from the 5th, 10th, 15th, 20th, 25th, and 30th generations were diluted with sterile distilled water to appropriate concentrations. A 100 μL aliquot of each dilution was spread on nutrient agar (NA) plates containing 300 μg/mL rifampicin and incubated at 32 °C for 14 h. The growth status of the RIF-resistant strains was observed, and 100 single colonies were randomly selected from the NA plates, transferred to fresh NA plates containing 300 μg/mL rifampicin, and incubated at 32 °C for 14 h. The number of colonies capable of normal growth was counted, and the percentage of the total was calculated to assess the genetic stability of rifampicin resistance [16,17,18]. The antagonistic ability of the original and RIF-resistant strains against *V. dahliae* was compared using the plate confrontation method, with the inhibition zone size indicating resistance activity [14].

### 2.4. Preparation of Antagonistic Bacteria and Pathogen Fermentation Broth

The RIF-resistant BHZ-29 strain, stored at −80 °C, was streaked onto NA plates, cultured, and activated overnight at 34 °C. A single colony was selected and inoculated into 100 mL of NB liquid medium, then shaken at 34 °C and 180 rpm for 24 h to obtain the antagonistic bacterial seed solution. This seed solution was inoculated at 2% into 250 mL of NB liquid medium and shaken at 32 °C and 180 rpm for 48 h to prepare the antagonistic bacterial fermentation broth [19,20].

A colony of *V. dahliae* preserved on a slant was picked with a sterile inoculation loop and spotted onto the center of a PDA plate. The pathogen plate was incubated at a constant temperature of 25 °C for 7–10 days. Fungal plugs were then punched from the plate using a puncher with an inner diameter of 6 mm. Fifteen fungal plugs were inoculated into 100 mL of Czapek’s liquid medium and shaken at 25 °C and 150 rpm for 2 days to obtain the pathogen seed culture. One milliliter of this seed culture was inoculated into 250 mL of Czapek’s liquid medium and shaken at 25 °C and 150 rpm for 5 days to prepare the pathogen fermentation broth [21].

### 2.5. Cotton Seedling Treatment

The colonization dynamics of strain BHZ-29 in cotton were characterized under gnotobiotic conditions to eliminate interference from indigenous soil microorganisms. The soil was sterilized at 180 °C for 3 h, and three varieties of cotton seeds were sown in the sterilized soil, with ten seeds per nutrient bowl, and grown in a greenhouse climate chamber. After germination, seedlings were thinned to six plants per pot. When the cotton seedlings developed two true leaves, the root irrigation method was used to inoculate them with a fermentation broth of antagonistic bacteria at a concentration of 2 × 10^8^ CFU/mL, applying 20 mL per plant. When the seedlings reached the stage of two leaves and one heart, they were inoculated with a fermentation broth of Verticillium dahliae at a concentration of 2 × 10^7^ spores/mL using the root injury perfusion method, with an inoculation volume of 20 mL per plant. Ten replicates (ten nutrient pots) were maintained for each treatment, with control (CK) seeds immersed in distilled water instead of the bacterial suspension. The experiment included four treatments: CK (blank control), BHZ-29 (inoculated with antagonistic bacteria), BHZ-29+VD (inoculated with antagonistic bacteria and pathogenic fungus) [14], and VD (inoculated with pathogens) [22,23]. Pots were kept in a growth chamber with day/night temperatures set at 30 °C/25 °C and a 12 h photoperiod with a light intensity of 200 μmol photons m^−2^ s^−1^. Soil moisture was maintained at 60% of the soil’s moisture-holding capacity.

### 2.6. Detection of Antagonistic Bacteria Colonization in Different Verticillium-Resistant Cotton Varieties

Ten cotton seedlings of different varieties were sampled on the 5th, 10th, 15th, 20th, and 25th days after inoculation with rifampicin-resistant BHZ-29. The ten cotton plants were divided into three biological replicates. Surface debris was washed off with tap water, and after natural drying, the root, stem, and leaf parts were weighed and surface-disinfected by treatment with 75% ethanol for 5 min, followed by rinsing five times with sterile distilled water. This was followed by treatment with 2% sodium hypochlorite (NaClO) for 3 min (2 min for leaves) and another five rinses with sterile distilled water. The final rinse solution (0.1 mL) was spread on nutrient agar (NA) plates and incubated at 32 °C; the absence of bacterial growth confirmed successful surface disinfection. The disinfected roots, stems, and leaves were dried with sterile filter paper, homogenized in a sterile mortar, and diluted with sterile distilled water. Serial dilutions (10^−1^, 10^−2^, 10^−3^) were plated (100 μL) on NA plates, with three replicates per dilution. After overnight incubation at 32 °C, single colonies were counted to determine the colonization dynamics of antagonistic bacteria in different cotton varieties [16,17].

### 2.7. Determination of Defense Enzyme Activity and Substance Content of Antagonistic Bacteria Against Different Varieties of Cotton

Ten cotton seedlings of different varieties were sampled on the 5th, 10th, 15th, 20th, and 25th days after inoculation. The ten cotton plants were divided into three biological replicates. Surface debris was washed off with tap water, and after natural drying, 0.1 g of fresh leaves was weighed and placed in an ice bath. In a mortar, 1 mL of the corresponding extraction buffer for POD, CAT, SOD, PPO, PAL, vitamin C (Vc), and malondialdehyde (MDA) was added, and homogenization was performed according to the manufacturer’s protocol. The test kits were purchased from Suzhou Keming Biotechnology Co., Ltd. in Suzhou, China.

### 2.8. Inoculation Procedures and Disease Evaluation

On the 25th day after inoculation, the number of diseased plants was recorded. Plants exhibiting obvious wilt or Verticillium wilt symptoms were assessed for disease severity using the grading scale described in reference [24]. The grading criteria are presented in Table 1. The disease index (DI) and control efficacy were calculated as follows:

Disease index = [∑ (number of plants per disease grade × corresponding grade value)/(total number of plants × highest grade value)] × 100%. Relative control effect (%) = [(control DI − treatment DI)/control DI] × 100%.

### 2.9. Statistical Analysis

The experiments were conducted using a completely randomized design. All experiments were performed in triplicate, and the results are presented as the mean ± standard deviation (SD). Statistical analyses were carried out using IBM SPSS software version 20.0. The antagonistic effect of bacteria was evaluated by one-way analysis of variance (ANOVA) to determine significant differences between treatments and controls. Mean values were compared using Duncan’s post hoc test at a significance level of *p* < 0.05.

## 3. Results

### 3.1. Genetic Stability and Resistance Activity of RIF-Resistant Strains

**Figure 1:**
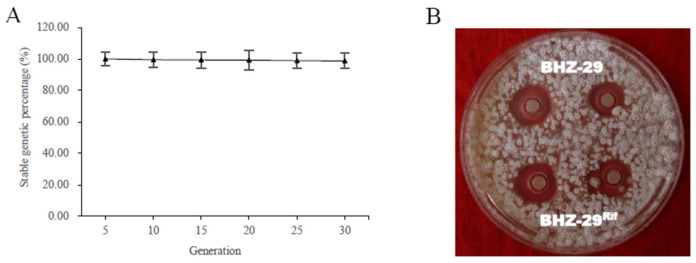
Detection of genetic stability (**A**) and antifungal activity against Verticillium dahliae (**B**) of BHZ-29RIF.

By gradually increasing the rifampicin concentration, RIF-resistant strains were obtained that grew normally on NA plates containing up to 300 μg/mL rifampicin, with no changes in colony morphology (Figure 1A). During continuous subculturing, 100 randomly selected colonies grew stably on NA plates with 300 μg/mL rifampicin, indicating no loss of rifampicin resistance in the RIF-resistant strains.

The plate confrontation assay showed that the inhibition zone size of the rifampicin-resistant strain was similar to that of the original strain, indicating that rifampicin labeling did not alter the antagonistic activity against *V. dahliae*. Thus, the labeled strain was suitable for subsequent colonization studies in cotton (Figure 1B).

### 3.2. Colonization Dynamics of Antagonistic Bacteria in Different Parts of Cotton

**Figure 2:**
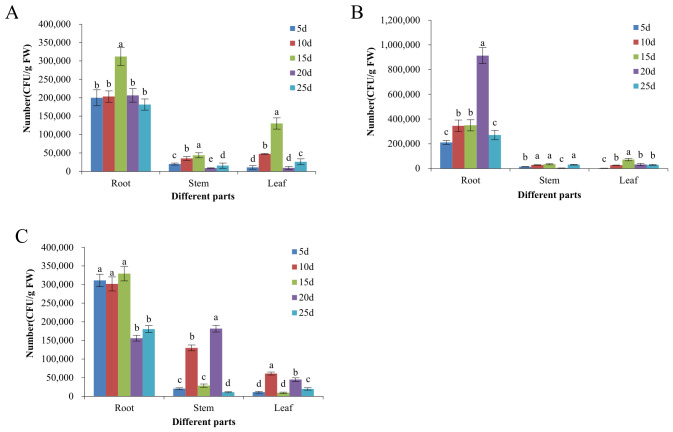
Colonization dynamics of antagonistic bacteria in different parts of cotton. (**A**) Xinluzao 36S (susceptible), (**B**) Xinluzao 61T, and (**C**) 9T (disease-resistant) cotton cultivars. Error bars represent the standard deviation of the mean of 3 replications. Values are expressed as means ± SD. Different lowercase letters indicate significant differences among treatments at *p* < 0.05 (Duncan’s test).

As shown in Figure 2, the colonization ability of BHZ-29 in the three cotton varieties followed the order root > stem > leaf, with colonization dynamics in all parts exhibiting an initial increase followed by a decrease. This spatial gradient suggests that the rhizosphere and root interior serve as the primary colonization niches for BHZ-29, likely due to the abundant supply of root exudates as carbon and energy sources. In contrast, the progressively lower bacterial populations in stems and leaves reflect the physical barriers and nutrient limitations encountered during endophytic translocation from belowground to aboveground tissues. Temporally, the colonization dynamics in all tested plant parts displayed a distinct “rise-and-decline” pattern over the sampling period. The initial ascending phase indicates successful establishment and active proliferation of BHZ-29 after inoculation, implying that the strain can effectively adapt to the plant’s internal environment.

Notably, significant variety-specific differences were observed in both the timing and magnitude of peak colonization. For instance, Xinluzao 61T reached its maximum root colonization considerably later than Xinluzao 36S and 9T, and its root-associated population density remained substantially higher throughout the experimental period. This delayed but enhanced root colonization suggests that Xinluzao 61T may provide a more favorable rhizosphere microenvironment or exhibit weaker early immune exclusion against BHZ-29, potentially allowing for a more sustained biocontrol effect. In contrast, the earlier peak of BHZ-29 in the leaves of variety 9T indicates a faster systemic translocation capacity, which might correlate with the specific vascular architectural traits of this cultivar.

### 3.3. Colonization Dynamics of Antagonistic Bacteria in Different Cotton Varieties Resistant to V. dahliae

**Figure 3:**
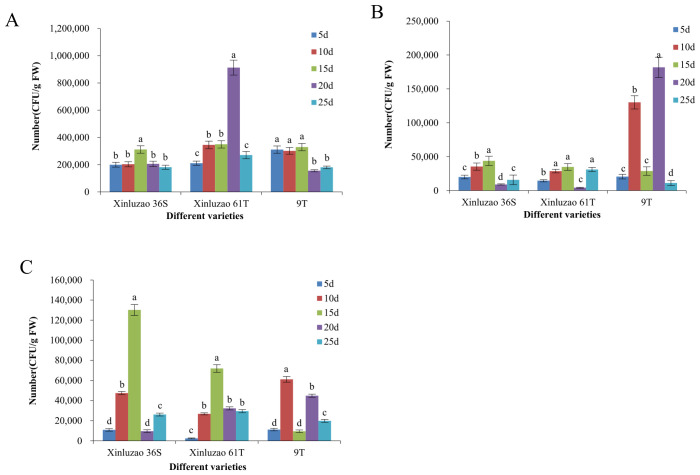
Colonization dynamics of antagonistic bacteria in different resistant cotton varieties. (**A**) Root, (**B**) stem, and (**C**) leaf. Error bars represent the standard deviation of the mean of 3 replications. Values are expressed as means ± SD. Different lowercase letters indicate significant differences among treatments at *p* < 0.05 (Duncan’s test).

As shown in Figure 3, BHZ-29 colonization exhibited clear variety-dependent and tissue-specific patterns across the three cotton cultivars. In roots, Xinluzao 61T supported the highest population, suggesting a more favorable rhizosphere environment or weaker constitutive root defenses. Its delayed peak implies slower but more sustained establishment, which is advantageous for long-term protection against soil-borne pathogens. In stems, however, variety 9T outperformed the others, indicating that stem colonization is governed by distinct host factors—such as vascular anatomy or translocation efficiency—independent of root abundance. In leaves, Xinluzao 36S showed the highest titers, whereas 9T exhibited the earliest peak, pointing to faster systemic transport in that cultivar. Temporally, all tissues displayed a rise-and-decline pattern, reflecting initial successful invasion followed by host-imposed regulation—likely through induced defenses or nutrient competition—to curb bacterial overgrowth. The timing and amplitude of this regulation varied among varieties, revealing differential host–microbe equilibrium points. Collectively, these findings underscore that biocontrol efficiency is cultivar-dependent: varieties with strong root colonization (e.g., Xinluzao 61T) may offer superior basal resistance, whereas those with enhanced stem and leaf colonization (e.g., 9T) could be more effective in systemic aerial protection. This highlights the need to match specific biocontrol strains with suitable cotton genotypes to optimize induced resistance, providing a mechanistic basis for integrated disease management.

### 3.4. Effect of BHZ-29 on Defense Enzyme Activities of Different Resistant Cotton Varieties

**Figure 4:**
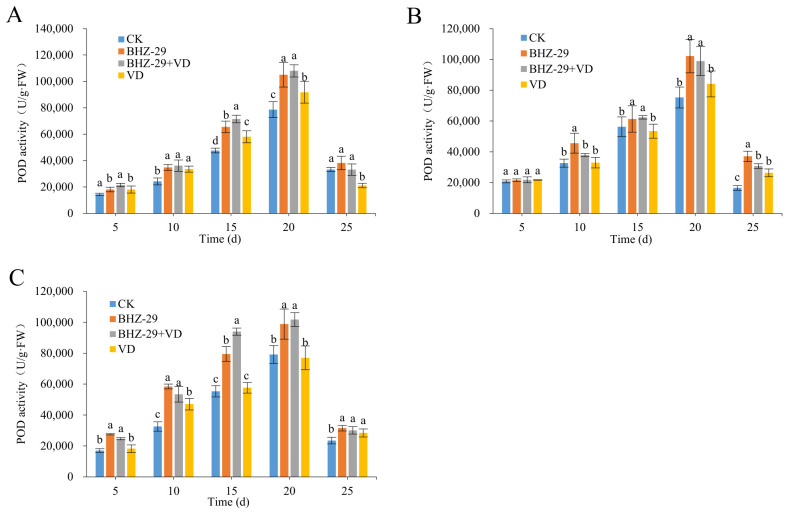
Effect of BHZ-29 on POD activity of different resistant varieties. (**A**) Xinluzao 36S (susceptible), (**B**) Xinluzao 61T, and (**C**) 9T (disease-resistant) cotton cultivars. Error bars represent standard deviation of mean of 3 replications. Values are expressed as means ± SD. Different lowercase letters indicate significant differences among treatments at *p* < 0.05 (Duncan’s test).

As shown in Figure 4, POD activity in all treatments exhibited a typical rise-and-fall pattern over time, with maximal levels reached at the same sampling point. In both Xinlu-zao 36S and Xinluzao 61T, the combined BHZ 29 + VD treatment consistently induced the highest POD activity, substantially exceeding that of the VD-only control, while BHZ 29 alone also elevated POD activity above the pathogen-only treatment, albeit to a lesser extent. This pattern indicates that BHZ 29 primes the cotton defense system, and subsequent pathogen infection further amplifies the enzymatic response. The comparable magnitude of induction across the two varieties suggests that BHZ 29’s eliciting effect is largely genotype-independent. Given that POD is a key enzyme in lignin biosynthesis and reactive oxygen species scavenging, its increased activity likely contributes to reinforced cell wall barriers and reduced oxidative damage, thereby restraining *V. dahliae* proliferation. Collectively, these findings demonstrate that BHZ 29 enhances cotton resistance not only through direct antagonism but also via systemic induction of defensive enzymes, with POD serving as a reliable indicator of the biocontrol-mediated immune response.

**Figure 5:**
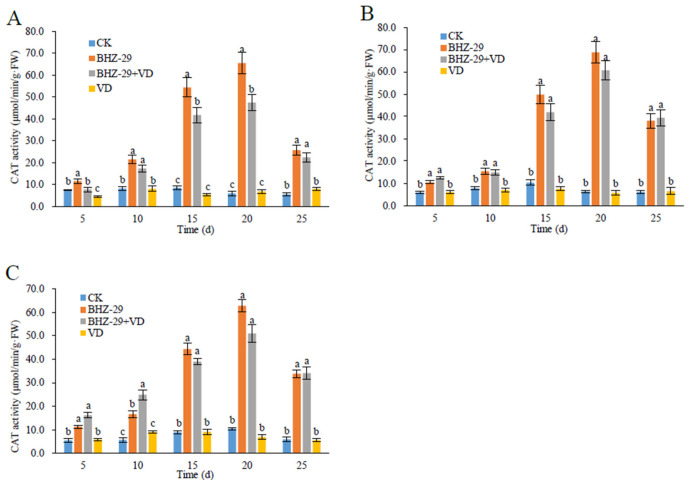
Effect of BHZ-29 on CAT activity of different resistant varieties. (**A**) Xinluzao 36S (susceptible), (**B**) Xinluzao 61T, and (**C**) 9T (disease-resistant) cotton cultivars. Error bars represent standard deviation of mean of 3 replications. Values are expressed as means ± SD. Different lowercase letters indicate significant differences among treatments at *p* < 0.05 (Duncan’s test).

As shown in Figure 5, catalase (CAT) activity in all varieties peaked at the same sampling point. Inoculation with BHZ 29 alone consistently elicited the highest CAT levels across cultivars, while the combined BHZ 29 + VD treatment also increased CAT activity compared to the pathogen-only control, though to a lesser extent. This suggests that BHZ 29 effectively primes the cotton antioxidant system, enhancing hydrogen peroxide (H_2_O_2_) scavenging capacity to mitigate oxidative damage during infection. Notably, Xinluzao 61T exhibited the strongest induction, indicating genotype-specific responsiveness. Given CAT’s role in protecting membrane integrity, this BHZ 29-mediated upregulation likely contributes to reducing cellular peroxidation and reinforcing overall disease tolerance.

**Figure 6:**
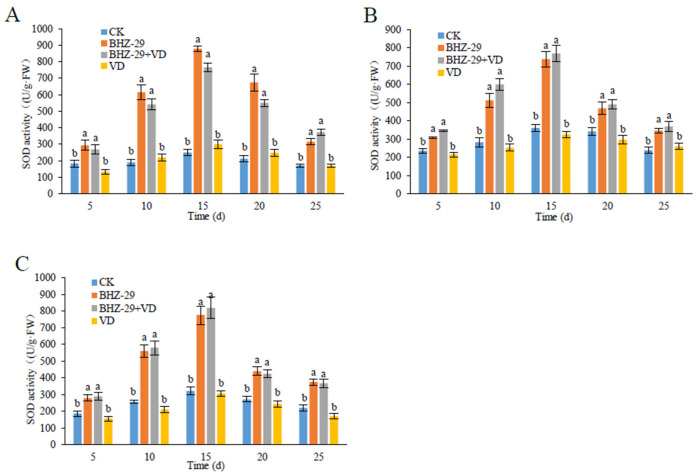
Effect of BHZ-29 on SOD activity of different resistant varieties. (**A**) Xinluzao 36S (susceptible), (**B**) Xinluzao 61T, and (**C**) 9T (disease-resistant) cotton cultivars. Error bars represent standard deviation of mean of 3 replications. Values are expressed as means ± SD. Different lowercase letters indicate significant differences among treatments at *p* < 0.05 (Duncan’s test).

As shown in Figure 6, SOD activity across the three cotton varieties exhibited a consistent temporal pattern of rise and decline. Inoculation with BHZ 29, either alone or in combination with VD, consistently elicited the highest SOD levels, while the pathogen-only (VD) treatment and untreated controls showed the lowest levels. This confirms that BHZ 29 effectively activates the cotton antioxidant system. Notably, variety-specific responses were observed: Xinluzao 36S showed the strongest induction under BHZ 29 alone, whereas Xinluzao 61T and 9T responded more robustly to the combined BHZ 29 + VD treatment, indicating that host genotype modulates the mode of defense priming. The elevated SOD activity is biologically significant, as this enzyme scavenges superoxide anions, thereby mitigating membrane lipid peroxidation and preserving cellular integrity under oxidative stress during pathogen attack. The enhanced response in the combined treatment further suggests that BHZ 29 primes the plant, and subsequent pathogen challenge amplifies the antioxidant defense, collectively contributing to improved tolerance against Verticillium wilt. These findings underscore that BHZ 29 reinforces cotton resistance not only through direct antagonism but also by orchestrating a genotype-dependent antioxidative response.

**Figure 7:**
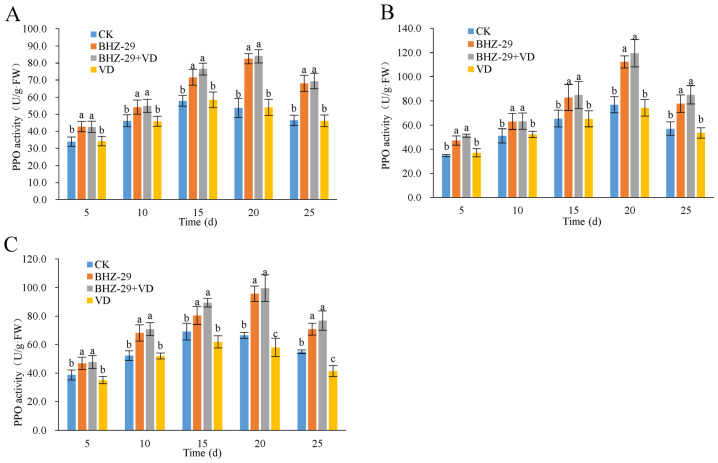
Effect of BHZ-29 on PPO activity of different resistant varieties. (**A**) Xinluzao 36S (susceptible), (**B**) Xinluzao 61T, and (**C**) 9T (disease-resistant) cotton cultivars. Error bars represent standard deviation of mean of 3 replications. Values are expressed as means ± SD. Different lowercase letters indicate significant differences among treatments at *p* < 0.05 (Duncan’s test).

As shown in Figure 7, the order of PPO activity across the three cotton varieties was consistently BHZ 29 + VD > BHZ 29 > CK > VD. The combined treatment elicited the highest PPO levels, whereas the pathogen-only treatment showed the lowest, with BHZ 29 alone and the untreated control exhibiting intermediate levels. This pattern reveals that BHZ 29 primes the plant’s defense system, and subsequent pathogen challenge further amplifies the enzymatic response. PPO is a critical enzyme in the phenylpropanoid pathway, oxidizing phenolics to quinones, which serve as precursors for lignin and phytoalexin synthesis. Its elevated activity strengthens cell wall barriers and restricts fungal hyphal expansion. Notably, this induction was observed uniformly across all cultivars, indicating that BHZ 29’s eliciting effect on PPO is largely genotype-independent. The coordinated upregulation of PPO, alongside other defense enzymes, underpins the systemic resistance conferred by BHZ 29, enhancing cotton’s overall tolerance to Verticillium wilt.

**Figure 8:**
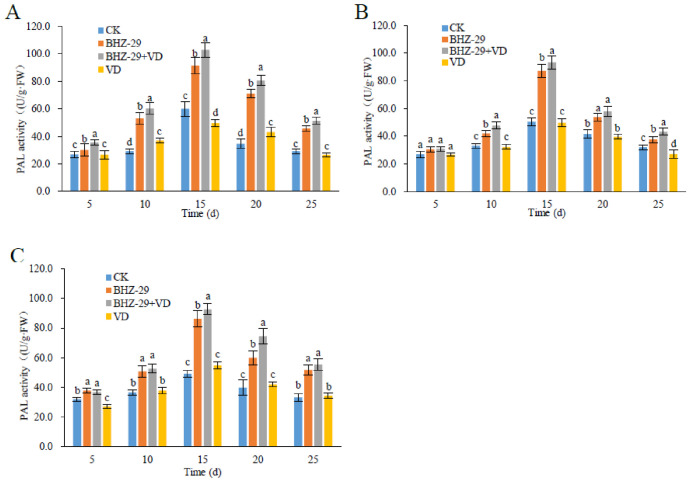
Effect of BHZ-29 on PAL activity of different resistant varieties. (**A**) Xinluzao 36S (susceptible), (**B**) Xinluzao 61T, and (**C**) 9T (disease-resistant) cotton cultivars. Error bars represent standard deviation of mean of 3 replications. Values are expressed as means ± SD. Different lowercase letters indicate significant differences among treatments at *p* < 0.05 (Duncan’s test).

As shown in Figure 8, PAL activity in all three cultivars exhibited a typical rise-and-fall pattern, with the maximum consistently observed at the same sampling point. Across all varieties, the combined BHZ 29 + VD treatment induced the highest PAL levels, while BHZ 29 alone also elevated activity above that of the pathogen-only treatment and untreated controls, which remained the lowest. This hierarchy demonstrates that BHZ 29 primes the phenylpropanoid pathway, and subsequent pathogen challenge synergistically amplifies enzymatic output. PAL catalyzes the first committed step in the biosynthesis of lignin, flavonoids, and phytoalexins; thus, its upregulation directly reinforces physical barriers and antimicrobial compound production. Notably, although all cultivars responded positively to BHZ 29, the magnitude of induction varied slightly, suggesting mild genotype-dependent modulation. The eventual decline in PAL activity after the peak likely reflects host-induced negative feedback to prevent excessive metabolic costs. Collectively, these findings confirm that BHZ 29 effectively enhances cotton’s systemic resistance via coordinated PAL activation, contributing to improved defense against Verticillium wilt.

### 3.5. Effect of BHZ-29 on Vc Content of Different Resistant Varieties

**Figure 9:**
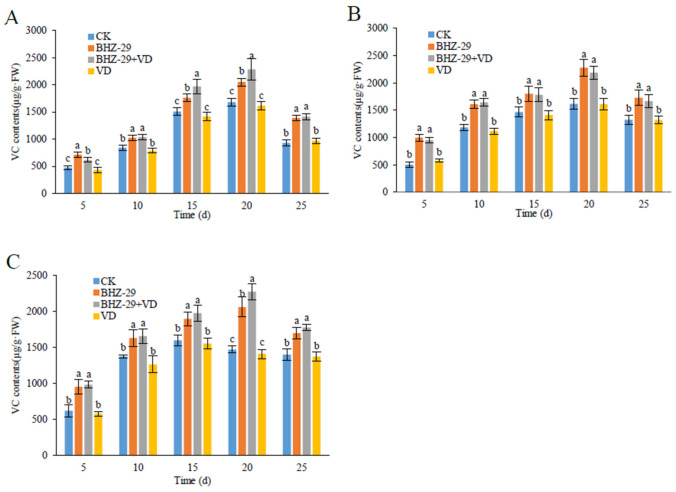
Effect of BHZ-29 on Vc content of different resistant varieties. (**A**) Xinluzao 36S (susceptible), (**B**) Xinluzao 61T, and (**C**) 9T (disease-resistant) cotton cultivars. Error bars represent standard deviation of mean of 3 replications. Values are expressed as means ± SD. Different lowercase letters indicate significant differences among treatments at *p* < 0.05 (Duncan’s test).

As shown in Figure 9, the vitamin C (Vc) content across the three varieties exhibited a consistent rise-and-fall pattern, peaking at the same time point. In all cultivars, treatments containing BHZ 29 (either alone or combined with VD) consistently produced the highest Vc levels, whereas the pathogen-only treatment and the untreated control remained significantly lower. This hierarchy indicates that BHZ 29 effectively stimulates the plant’s antioxidant pool, and subsequent pathogen challenge further amplifies this accumulation. Vc, as a critical non-enzymatic antioxidant, directly neutralizes reactive oxygen species, thereby mitigating membrane lipid peroxidation and preserving cellular integrity under oxidative stress. Although the magnitude varied slightly among varieties, the overall induction was robust across genotypes, suggesting that BHZ 29’s effect on Vc is largely genotype-independent. The eventual decline after the peak likely reflects a regulatory mechanism to maintain redox homeostasis. Collectively, BHZ 29 reinforces cotton’s defense not only through enzymatic antioxidants but also by elevating Vc, enhancing systemic tolerance to Verticillium wilt.

### 3.6. Effect of BHZ-29 on MDA Content of Different Resistant Varieties

**Figure 10:**
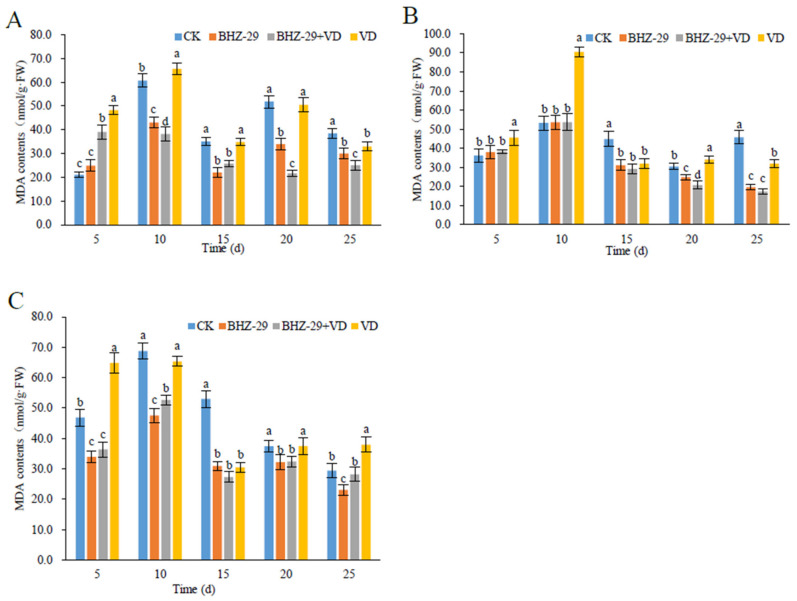
Effect of BHZ-29 on MDA content of different resistant varieties. (**A**) Xinluzao 36S (susceptible), (**B**) Xinluzao 61T, and (**C**) 9T (disease-resistant) cotton cultivars. Error bars represent standard deviation of mean of 3 replications. Values are expressed as means ± SD. Different lowercase letters indicate significant differences among treatments at *p* < 0.05 (Duncan’s test).

As shown in Figure 10, MDA content in all three varieties followed a rise-and-fall trajectory, with treatment rankings consistently as VD > CK > BHZ 29 > BHZ 29 + VD across cultivars. Pathogen inoculation alone caused the highest MDA accumulation, reflecting extensive membrane lipid peroxidation and cellular damage. In contrast, BHZ 29 inoculation—especially when combined with VD—markedly reduced MDA levels, indicating effective protection against oxidative stress. This reduction is attributable to BHZ 29-mediated upregulation of antioxidant enzymes and non-enzymatic scavengers, which quench excess reactive oxygen species and maintain membrane stability. Although the magnitude and timing of MDA changes differed among Xinluzao 36S, 61T, and 9T, the consistent ranking underscores a genotype-independent protective effect of BHZ 29. The post-peak decline suggests that active repair mechanisms are engaged to restore cellular homeostasis. Collectively, these findings demonstrate that BHZ 29 alleviates oxidative membrane injury, reinforcing cotton’s systemic tolerance to Verticillium wilt beyond direct pathogen suppression.

### 3.7. Effect of Antagonistic Bacteria on Cotton Verticillium Wilt

As shown in Table 2, no disease symptoms were observed in the untreated control across all three varieties, confirming the absence of natural infection. Inoculation with *V. dahliae* alone resulted in severe disease development in every cultivar, whereas pretreatment with BHZ 29 significantly reduced the disease index in all cases, demonstrating a consistent protective effect of the biocontrol strain. Notably, the magnitude of disease reduction varied among cultivars: the highest control efficacy was achieved in Xinluzao 36S, followed by 9T, with the lowest in Xinluzao 61T. This graded response closely paralleled the inherent resistance levels of the varieties, suggesting that BHZ 29-mediated protection is amplified when combined with stronger host genetic resistance. Conversely, in more susceptible backgrounds, the biocontrol effect was partially attenuated, likely due to greater pathogen pressure overwhelming the induced defense responses. These findings imply that BHZ 29 does not confer absolute immunity but rather enhances the existing resistance framework of the plant. The synergistic interaction between exogenous biocontrol and intrinsic varietal resistance underscores the importance of selecting appropriate cotton genotypes to maximize integrated disease management outcomes against Verticillium wilt.

## 4. Discussion

Biological control of soil-borne cotton Verticillium wilt has recently garnered increased global interest. Regarding the mechanism by which antagonistic bacteria control plant diseases, previous studies suggest that these bacteria primarily exert inhibitory effects and compete at infection sites [25,26]. Therefore, ideal biocontrol antagonistic bacteria should possess not only strong antagonistic activity and a broad antibacterial spectrum but also robust viability and colonization capacity within host plants. Although screened bacteria often show positive results in artificially prepared media, their growth and function under natural conditions can be impeded by competition from soil microorganisms or other adverse environmental factors, resulting in variable outcomes. Thus, to ensure stable and effective disease control in production, it is essential to advance research on colonization theory.

When using endophytic bacteria to control soil-borne diseases, effective colonization within plants is a crucial prerequisite for biocontrol efficacy. Therefore, studying the colonization ability of endophytic bacteria has become an important focus of current research on plant protection mechanisms [27,28,29,30]. In this study, traditional rifampicin resistance labeling was employed to recover and detect the colonization of BHZ-29R in cotton plants following root irrigation. The results showed that BHZ-29 could colonize cotton plants, with higher colonization levels and longer persistence in roots compared to stems. However, colonization in both roots and stems gradually decreased over time, eventually reaching low levels. The population of BHZ-29R in cotton roots and stems remained relatively stable during the first five days after inoculation. Reinoculation after this period to maintain a certain population advantage might provide long-term protection for plants. The relationship between colonization level, inoculation amount, and the control efficacy of this strain requires further investigation. In this study, rifampin-resistant strains were used to confirm that endophytic bacteria BHZ-29 could colonize in tissues such as the roots, stems and leaves of cotton plants, but this method could not determine the spatial location of bacteria in plant tissues. This is a limitation of this study. The next step is to use GFP to label the endophyte BHZ-29, allowing us to determine the spatial location of colonization.

Research has shown that plants can specifically attract microorganisms beneficial to their ecology and evolution. Just as rhizosphere bacteria are selectively recruited, endophytic bacteria are also selectively colonized within plants. Not all soil bacteria can enter plants and become endophytes [31]. During the screening of biocontrol bacteria against watermelon Fusarium wilt, we isolated a strain, SHT-2, from soil which exhibited strong antagonistic activity and effective control in pot experiments. However, when tested using rifampicin resistance marker technology, the marked strain was not detected in cotton roots and stems, indicating its inability to colonize cotton plants. Previous studies have suggested that the biocontrol bacterium *Paenibacillus polymyxa* C5 can form biofilms on tobacco root surfaces but cannot penetrate root tissues [32]. Plants act as filters for soil microorganisms, selecting capable endophytic bacteria [32], but the specific mechanisms involved are complex and not yet fully understood.

Antagonistic bacteria not only effectively colonize cotton but also induce systemic resistance. The results of this study showed that enzyme activities in resistant varieties were higher than those in susceptible varieties. In recent years, the role of biocontrol bacteria in inducing plant disease resistance has attracted significant attention. Studies have demonstrated that protective enzymes such as superoxide dismutase (SOD), peroxidase (POD), catalase (CAT), polyphenol oxidase (PPO), and phenylalanine ammonia-lyase (PAL), which are involved in various physiological and metabolic processes in plants, are closely related to plant defense responses and disease resistance. These enzymes are often used as important indicators to assess plant defense mechanisms [8]. PAL and PPO are associated with the formation of phenolic compounds and lignin in plants and serve as key regulatory enzymes for systemic resistance. POD and CAT are closely involved in scavenging reactive oxygen species in plants and are positively correlated with disease resistance [33,34].

The content of malondialdehyde (MDA), the final product of membrane lipid peroxidation, reflects the extent of membrane lipid peroxidation and cell membrane damage, showing a positive correlation [35,36]. Ascorbic acid (Vc) is a plant antioxidant that can directly scavenge superoxide anions (O_2_^−^) and hydroxyl radicals (·OH), and convert hydrogen peroxide (H_2_O_2_) into water via ascorbic acid oxidase. When plants are damaged by pathogens or stress factors, Vc reduces the toxicity of reactive oxygen species and helps maintain the normal physiological functions of plant cells [37,38,39].

In this study, RIF-resistant BHZ-29 was obtained using the antibiotic labeling method, and its colonization in the roots, stems, and leaves of different resistant cotton varieties was investigated. The results showed that BHZ-29 effectively colonized these tissues, with colonization ability ranked as root > stem > leaf. The maximum root colonization was 9.13 × 10^5^ CFU/g in Xinluzao 61T on day 20, the maximum stem colonization was 1.82 × 10^5^ CFU/g in 9T on day 20, and the maximum leaf colonization was 1.30 × 10^5^ CFU/g in Xinluzao 36S on day 15. These findings indicate that BHZ-29 can not only enter and survive in cotton roots via initial root inoculation but also move upward with plant growth, stably colonizing various parts to exert biocontrol effects. This is consistent with previous studies on the colonization characteristics of antagonistic bacteria [16,17,18,38,39]. Gu et al. found that *Bacillus amyloliquefaciens* Lj1 could affect defense enzyme activities in cucumber plants and induce resistance against powdery mildew [40]. A consistent physiological response was detected in the present study: both single inoculation with BHZ-29 and co-inoculation of BHZ-29 combined with VD markedly elevated the activities of POD, CAT, SOD, PPO and PAL as well as the vitamin C content in cotton, whereas the MDA concentration was significantly reduced. These physiological changes suggest that BHZ-29 is capable of activating cotton defense responses. Nevertheless, further pathogenicity and pot trials are required to verify its practical control efficacy against cotton Verticillium wilt. Collectively, strain BHZ-29 exhibits promising potential as a biocontrol agent for this soil-borne disease, and our findings lay a theoretical foundation for the subsequent development and field application of antagonistic biocontrol bacteria.

This study only conducted relevant physiological and control efficacy experiments under controlled greenhouse conditions, which inevitably carries certain limitations. Greenhouse environments feature stable temperature, humidity and soil conditions, isolated from complex field interferences such as variable weather, diverse soil microflora, mixed weed communities and natural pathogen pressure. Consequently, the colonization capacity, induced resistance effect and disease control performance of the BHZ-29 biocontrol agent observed in pots may fail to be fully replicated under actual agricultural production conditions. To accurately evaluate the field adaptability and sustained control efficiency of strain BHZ-29 before large-scale popularization and application, systematic multi-site field verification trials are urgently required in follow-up research. This work shows the protective effect of strain BHZ-29, and the next step is to focus on analyzing whether *Bacillus velezensis* BHZ-29 can produce antibacterial metabolites, lipopeptides, siderophores, hydrolases and other factors.

## 5. Conclusions

In conclusion, BHZ 29 demonstrates promising potential as a biocontrol agent for sustainable cotton production by effectively activating systemic defense responses across diverse cultivars. Its consistent protective effects, although influenced by host resistance levels, highlight the feasibility of integrating microbial inoculants with varietal selection for optimized disease management. The strain’s ability to prime both enzymatic and non-enzymatic antioxidant systems offers a multifaceted strategy to mitigate Verticillium wilt, thereby reducing dependence on chemical fungicides. Future efforts should focus on formulating stable inoculants and validating field performance under varying agroecological conditions, translating these laboratory findings into practical, eco-friendly solutions for cotton growers and contributing to the broader adoption of green agricultural practices.

## Figures and Tables

**Table 1:** Classification system for disease rating of cotton Verticillium wilt.

Disease Grade	Grading Standard
0	no symptoms
1	leaf loss and/or less than 25% of leaves showing strong wilt symptoms
2	leaf loss and/or 25–50% of leaves showing strong wilt symptoms
3	leaf loss and/or 50–75% of leaves showing strong wilt symptoms
4	leaf loss exceeding 75% or total leaf loss

**Table 2:** Disease index and relative control efficacy of cotton Verticillium wilt treated with antagonistic bacteria.

Treatment	Varieties	Disease Index	Biocontrol Efficacy (%)
CK	36S	0.00 ± 0.00	
BHZ-29	36S	0.00 ± 0.00	
BHZ-29+VD	36S	40.74 ± 0.85 d	57.55 ± 0.96 c
VD	36S	95.96 ± 1.66 a	
CK	61T	0.00 ± 0.00	
BHZ-29	61T	0.00 ± 0.00	
BHZ-29+VD	61T	23.29 ± 0.72 f	72.04 ± 1.37 a
VD	61T	83.29 ± 1.53 b	
CK	9T	0.00 ± 0.00	
BHZ-29	9T	0.00 ± 0.00	
BHZ-29+VD	9T	29.64 ± 0.68 e	61.82 ± 1.01 b
VD	9T	77.65 ± 1.47 c	

Note: Different letters indicate statistically significant differences between treatments (least significant difference test; *p* < 0.05). CK, blank control; BHZ-29, inoculated with antagonistic bacteria; BHZ-29+VD, inoculated with antagonistic bacteria + pathogenic fungus; and VD, inoculated with pathogens.

## Data Availability

The datasets generated in this study are available upon request from the corresponding author.
